# Host-switching by a vertically transmitted rhabdovirus in *Drosophila*

**DOI:** 10.1098/rsbl.2011.0160

**Published:** 2011-03-30

**Authors:** Ben Longdon, Lena Wilfert, Jewelna Osei-Poku, Heather Cagney, Darren J. Obbard, Francis M. Jiggins

**Affiliations:** 1 Institute of Evolutionary Biology, and Centre for Immunity, Infection and Evolution, University of Edinburgh, Edinburgh EH9 3JT, UK; 2 Department of Genetics, University of Cambridge, Cambridge CB2 3EH, UK

**Keywords:** rhabdovirus, *Drosophila*, host-shift, sigma virus, phylogeny, vertically transmitted

## Abstract

A diverse range of endosymbionts are found within the cells of animals. As these endosymbionts are normally vertically transmitted, we might expect their evolutionary history to be dominated by host-fidelity and cospeciation with the host. However, studies of bacterial endosymbionts have shown that while this is true for some mutualists, parasites often move horizontally between host lineages over evolutionary timescales. For the first time, to our knowledge, we have investigated whether this is also the case for vertically transmitted viruses. Here, we describe four new sigma viruses, a group of vertically transmitted rhabdoviruses previously known in *Drosophila*. Using sequence data from these new viruses, and the previously described sigma viruses, we show that they have switched between hosts during their evolutionary history. Our results suggest that sigma virus infections may be short-lived in a given host lineage, so that their long-term persistence relies on rare horizontal transmission events between hosts.

## 1. Introduction

Many animals have intimate associations with protists, bacteria and viruses, which live within the cytoplasm of their cells and are transmitted vertically between generations [1]. Vertical transmission and an inability to survive for long outside of the host mean that endosymbionts might be expected to show extreme host-fidelity and cospeciate with their hosts. Indeed, phylogenies of bacterial endosymbionts show obligate mutualists have remarkably stable associations with their hosts. For example, *Buchnera* bacteria, which synthesize amino acids lacking from the diet of aphids, have been stably vertically transmitted for approximately 150–250 Myr [2]. Similar patterns have been found in other mutualists such as *Wigglesworthia* in tsetse flies [3], *Blochmannia* in carpenter ants [4] and *Blattabacterium* in cockroaches and termites [5]. By contrast, parasitic endosymbionts persist for relatively short periods in a given host lineage and frequently switch host species. For example, there is little or no congruence between the phylogenies of *Wolbachia*, [6], *Rickettsia* [7] and *Spiroplasma* bacteria [8] and their arthropod hosts. These associations may be unstable as hosts can evolve resistance and drive the parasite to extinction [9].

In contrast to bacterial endosymbionts, little is known about the evolutionary history of vertically transmitted viruses. Sigma viruses are vertically transmitted rhabdoviruses previously known from three species of *Drosophila*—*Drosophila melanogaster* (DMelSV) [10], *Drosophila obscura* (DObsSV) and *Drosophila affinis* (DAffSV) [11]. These viruses are unusual in that they are transmitted vertically through both eggs and sperm [10,12]. Here, we describe four new sigma viruses that each infect a different species of *Diptera*, and use a phylogenetic approach to show that sigma viruses have switched between host species during their evolution.

## 2. Material and methods

### (a) Viral discovery and sequencing

We collected *Drosophila tristis* in Derbyshire, UK; *Drosophila immigrans* in Marktredwitz, Germany; *Drosophila ananassae* in Kilifi, Kenya; and *Muscina stabulans* in Cambridge, UK. Infected flies were detected by exposing them to pure CO_2_ at 12°C for 15 mins. Uninfected flies recover after approximately 30 mins while infected flies remain paralysed [10]. RNA was extracted from paralysed flies, reverse transcribed (see [11]), and amplified by PCR using multiple degenerate primers targeted to conserved regions of the viral RNA-dependent RNA polymerase gene (RDRP) (electronic supplementary material, table S1). PCR products were sequenced using BigDye reagents (GenePool facility, University of Edinburgh, UK) and once a small region of the RDRP gene had been sequenced, 3′ RACE (rapid amplification of cDNA ends) was used to obtain further sequence (see [11]). To obtain high-quality sequences, new primers were designed to amplify the fragment sequenced by RACE, and this was re-sequenced in both directions. The host species was confirmed by sequencing mitochondrial *COI* and/or *Cytb* genes. Additional species were also collected and tested with the CO_2_ assay, but we only report those species from which we were able to amplify a sigma virus.

### (b) Inferring the virus phylogeny

The nucleotide sequence of the RDRP genes from sigma viruses and other rhabdoviruses was aligned based on the translated amino acid sequence using ClustalW. Alignments were trimmed to contain only a conserved region of the RDRP that could be robustly aligned. Phylogenies were inferred using maximum-likelihood (ML) (PAUP [13]) and Bayesian (MrBayes [14]) methods. The ML analysis used a heuristic search with a nearest neighbour interchange algorithm and a general time reversible model with a gamma-distributed rate variation and a proportion of invariable sites. This model of sequence evolution was selected by comparing alternative models using Akaike information criterion in Model Test [15]. Node-support was estimated by non-parametric bootstrapping. The Bayesian analysis used the same model of sequence evolution and the Markov chain Monte Carlo was run for 1 million generations, sampled every 100 steps with the first 25 per cent of samples being discarded as burn-in.

### (c) Detecting incongruent tree topologies

To detect topological incongruence between host and parasite phylogenies, we used a Shimodaira–Hasegawa test (SH-test) [16], which compares the likelihood of the viral phylogeny inferred from the data with one constrained to match the host topology [17,18]. We also used a Bayesian approach that identifies the proportion of the posterior sample of viral topologies that match the host phylogeny (e.g. [19]). As these approaches compare only topologies (and not branch lengths), they are a conservative test for host switching. Even when topologies are incongruent, some cospeciation or switching between related hosts may make host and virus topologies more similar than expected by chance. To test for topological similarity, we compared the distribution of Robinson-Foulds [20] distance metrics provided by 10^4^ random viral topologies to that derived from the posterior sample of viral topologies.

## 3. Results

We detected novel sigma viruses in four dipteran species, including three species of *Drosophila*—*D. tristis*, *D. immigrans* and *D. ananassae*—and one member of the Muscidae, *Muscina stabulans*. We have tentatively named these new viruses as DTriSV, DImmSV, DAnaSV and MStaSV, respectively. This brings the total number of sigma viruses described to seven, and for the first time extends their distribution outside the genus *Drosophila*.

**Figure 1.**
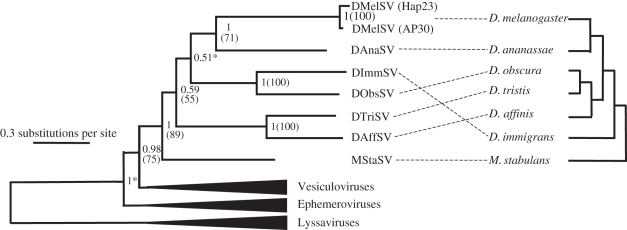
Bayesian phylogeny of the sigma viruses (left) and their hosts (right). Node labels represent Bayesian posterior supports with maximum-likelihood bootstrap support in brackets. The tree is rooted with the Lyssavirus clade. Non-sigma virus clades are collapsed. Nodes marked with an asterisk had bootstrap support of less than 50%. *Drosophila melanogaster* and *D. ananassae* shared a common ancestor approximately 20 million years ago (Ma) but both fall within the *D. melanogaster* group, which is separated from the *obscura* group (*D. obscura, D. affinis* and *D. tristis*) by approximately 25 Ma. Both of these groups fall within the subgenus *Sophophora*, while *D. immigrans* is in the subgenus *Drosophila*, which separated from *Sophophora* approximately 40 Ma (dates from [21]).

We sequenced 1845–3006 bp of the RDRP gene from the four viruses (accession-numbers: JF311399–JF211402). The sequences are highly divergent from one another, with the most closely related pair, DAffSV and DTriSV, having an amino acid sequence identity of 0.73. The other five genes of sigma viruses have previously been shown to have even greater divergence [11]. The new sigma viruses form a clade of dipteran-infecting viruses that also contains the previously described sigma viruses DMelSV, DObsSV and DAffSV (figure 1). In common with previous phylogenies [11,22], the sigma virus clade is most closely related to the Ephemerovirus and Vesiculovirus clades (which together form the Dimarhabdoviruses).

To test whether the sigma viruses have exclusively cospeciated with their hosts, we compared the host and virus phylogenies. The phylogeny of these host species is extremely well resolved [17,18]. We found the likelihood of the virus tree constrained to follow the topology of the host taxa was significantly reduced (SH-test: 2*ΔlnL* = 328, *p* < 0.005). For the Bayesian trees, we also found that the viral phylogeny differed significantly from the host topology, with none of the topologies in the posterior sample of trees matching that of the hosts. Therefore, both methods suggest these viruses have switched between host species. Incongruence is owing to two factors: first, the presence of DImmSV in a clade of viruses with hosts from a different subgenus of *Drosophila* to *D. immigrans* [18]; and also *D. obscura* is much more closely related to *D. tristis* than *D. affinis* [17], yet a viral clade comprising DTriSV and DAffSV is well-supported.

However, although the trees were not congruent, we found that the inferred virus topology was more similar to the host topology than expected by chance. Only 2 per cent of random viral topologies were closer to the host topology than the posterior sample of actual virus topologies were to the host topology [20]. This may imply cospeciation events, but could be owing to other factors such as preferential host switching between closely related species.

## 4. Discussion

We have discovered four new sigma viruses in *D. ananassae*, *D. immigrans*, *D. tristis* and *M. stabulans*. Together with the three existing sigma viruses in other *Drosophila*, they form a clade of Dipteran-infecting rhabdoviruses. It is probable that these viruses are vertically transmitted as not only are all of the previously known sigma viruses vertically transmitted [12], but also vertical-transmission of CO_2_ sensitivity—the hallmark of sigma virus infection—is known from other *Diptera* [23,24]. The phylogeny of the viruses reflects neither the phylogeny of the hosts, nor the region of the world where they were collected (these viruses were isolated in Europe, Africa and America). Therefore, sigma viruses have switched between host lineages during their evolution.

Sigma viruses have highly dynamic interactions with their hosts. In *D. melanogaster* populations, there has been a recent selective sweep of a gene conferring resistance to DMelSV [25], and this was followed by the sweep of a viral genotype that overcomes host resistance [26]. DObsSV also shows evidence of a recent and rapid sweep [12]. Such rapid changes in host resistance are expected to drive fluctuations in viral prevalence, and may make virus–host associations unstable and short-lived [9]. If so, then the virus will only persist in the long-term by switching between host species. This appears to be a general phenomenon among vertically transmitted parasites, as similar patterns are seen among bacterial endosymbionts (see §1) [6–8], and genomic parasites such as transposable elements [27] and homing endonucleases [28]. Although the transfer mechanism is unclear, we have previously suggested that parasitic mites could act as vectors of sigma viruses [11], and arthropod vectors may be responsible for other endosymbionts and genomic parasites switching between host lineages [27,29,30].

A sigma-like virus outside of the genus *Drosophila* suggests that these viruses may be widespread in Dipterans, if not insects as a whole. Unlike bacterial endosymbionts, the rapid evolution of the sigma virus genome makes it impossible to design a single pair of diagnostic PCR primers that can be used to test for new strains of the virus. In the course of this study, we encountered CO_2_-sensitive individuals of other species of flies from which we were unable to amplify virus using our primers, and these may harbour other sigma-like viruses. CO_2_ sensitivity has also been reported in 13 other *Drosophila* species [10], and in *Culex* mosquitoes [23]. Additionally, rhabdovirus sequences have inserted into the genomes of various insect species [31] and rhabdovirus-like particles have been found in firebug testes [32]. The non-Drosophilid sigma virus we found is of particular interest, as the closely related Dimarhabdoviruses are vector-borne diseases of vertebrates (some of which are vectored by other dipterans) [22]. The discovery of other rhabdoviruses in insects that do not blood-feed may make it possible to understand how viruses may have switched between being vector-borne pathogens of vertebrates and being purely entomopathogenic.
